# *“Men are the head of the family*, *the dominant head”*: A mixed method study of male involvement in maternal and child health in a patriarchal setting, Western Nigeria

**DOI:** 10.1371/journal.pone.0276059

**Published:** 2022-10-26

**Authors:** Ifeoma Peace Okafor, Chioma Lilian Chukwudi, Ugonnaya Ugochinyere Igwilo, Babatunde Enitan Ogunnowo

**Affiliations:** Department of Community Health & Primary Care, College of Medicine, University of Lagos, Lagos Nigeria; South African Medical Research Council, SOUTH AFRICA

## Abstract

**Introduction:**

The importance of involving men in reproductive, maternal and child health (RMCH) programs is increasingly recognized globally. In Nigeria, most MCH services do not actively engage fathers.

**Aim:**

To assess men’s knowledge, involvement in MCH and the barriers in Southwest Nigeria. Predictors of good knowledge and involvement were also assessed.

**Methods:**

This was a community-based cross sectional study. Quantitative and qualitative methods were used in data collection which was done within a period of three months in 2018. Quantitative data were collected with interviewer administered questionnaires from 418 respondents who were selected by multistage sampling methodology. The topic was further explored using focus group discussion (FGD). Quantitative data were analysed using EPI-INFO version 7. Quantitative variables were summarized using means and standard deviations while multivariable analysis was carried out to determine predictors of good knowledge and involvement in MCH. A combination of deductive and inductive analysis was used for the qualitative data.

**Results:**

Overall, 65% of men had good knowledge of MCH while 60.8% had good involvement. Predictors of good knowledge were being a Christian (AOR 1.674, 95% CI 1.045–2.679), being of Yoruba tribe (AOR 1.753, 95% CI 1.100–2.796), having post-secondary education (AOR 1.984, 95% CI 1.002–3.928), having more under-fives in the household (AOR 2.162 95% CI 1.365–3.425) and spouse having post-secondary education (AOR 2.755, 95% CI 1.189–6.382). Predictors of good involvement in MCH include higher educational level of spouse: secondary (AOR 2.852, 95% CI 1.214–6.699), post-secondary (AOR 2.270, 95% CI 1.000–5.161) and having good knowledge of MCH (AOR 2.518, 95% CI 1.587–3.994). From the FGD, other factors which influence involvement were related to traditional/cultural orientation, time constraint and finance among others.

**Conclusion:**

Men’s knowledge and their involvement in maternal and child health were sub-optimal. For improvement, community-based intervention programmes should be designed for men and implemented, taking into consideration their traditional/cultural roles, religious orientation, busy schedules, and educational backgrounds. They should be re-oriented on their patriarchally informed belief about their perceived roles in RMCH.

## Introduction

Maternal and child health (MCH) care is a vital part of the health care system in any country. Mothers and children are vulnerable groups in the society and adverse social and economic conditions affect them most [1]. The United Nations in its Millennium Summit in 2000, adopted and dedicated goals 2–5 of the Millennium Development Goals to the wellbeing of mothers and children [2], and went further in the Sustainable Development Goal 3 (Agenda for 2030) to target the significant reduction in global maternal, newborn and child mortality [3].

Since the International Conference on Population and Development (ICPD) held in Cairo in 1994, there has been an increased recognition of the importance of involvement of men in reproductive, maternal and child health (RMCH) [4]. The conference highlighted the benefits that come with this and stressed that special efforts be made to promote their active involvement in areas such as: responsible parenthood, sexual and reproductive behavior including family planning, MCH, children’s education, health and nutrition, among others [5]. The importance of the involvement of men in MCH was further stressed in the World Health Organization (WHO) recommendations on health promotion interventions for maternal and newborn health [6]. Over time, studies have shown the benefits of male involvement in areas such as participation in maternity care, child care and household tasks, breastfeeding, maternal depression, PMTCT, among others [7–14].

Despite the recognized significant role of men in RMCH and the benefits therein, men still have very limited knowledge of these [7]. The deep-rooted social structures of developing societies have formed an inequity over the power of making decision on various aspects of reproductive health [15]. Furthermore, when men used to be the sole income generators of the family, their attention towards family health and their involvement in RH was limited [16]. This has led women to be the basic target in most of the health education and awareness programmes and men tend to be excluded [9]. This exclusion indicates a reason behind men being unable to make informed decisions during emergencies and being less interested to engage in RMNCH [17].

While the outlets of maternal health services are growing, lack of men’s support for the utilization of safe motherhood services is seen as a barrier by the women [18]. Historically, men’s engagement has been depicted as obstructive or nonexistent in RH matters. They either impede on women’s choice on the use of family planning methods or become nonresponsive due to lack of interest [19].

As regards the Nigerian culture, the patriarchal system is entrenched and gender roles have already been mapped out for men and women. Men should be the decision makers while women should be submissive and accept whatever decisions made by their husbands. It was up to the husbands they were to marry, not the women themselves, to make their lives successful and happy. Thus, they were robbed of every initiative and resourcefulness which could enable them make decisions affecting them and the family generally [20].

Male dominance can have negative effects on mother and baby. Their lack of participation at family planning, and antenatal and postnatal consultations means that they do not benefit much from any information given by health providers, or about their roles in them [21]. There is a need for holistic approach for the involvement of men in RH. If men and women are perceived as equal partners, effective decision making and better outcomes in MCH can be expected [22, 23]. It is against this background that this study was conducted to assess the factors affecting men’s knowledge and involvement in MCH care in southwest Nigeria. The results of this study will add to existing evidence to facilitate targeted interventions for improvements in MCH in similar settings.

## Materials and methods

### Description of study area

The study was conducted in Oshodi-Isolo, an urban Local Government Area (LGA) in Lagos State, southwest Nigeria. The LGA is the third and lowest tier of government in the country. The study LGA is part of the Ikeja Division of Lagos State. As at 2006, it had a population of 1,134,548 people, comprising 514,857 males and 619,691 females, with the majority between the ages of 15–64 years [24]. The 2019 projected population according to the Lagos State Abstract of Local Government Statistics was 1,698,565. The LGA is made up of people of varying educational, religious, occupational and ethnic backgrounds but mostly Yoruba tribe. In western Nigeria and the country as a whole, the patriarchal system is the norm [25].

The maternal mortality ratio of Oshodi-Isolo LGA is 443 per 100,000 [26]. Formal and informal health care services are available in the area. Most of the residents live within five kilometers from formal health facilities that provide MCH services.

### Study design

This was a community-based, cross sectional study employing qualitative and quantitative data collection methods.

### Study population

#### Quantitative and qualitative

The study population consisted of adult men living in the study area. Only those who were married or co-habiting, with at least one child below 5 years, and had lived in the study area for at least 6 months were included in the study.

### Sample size estimation

A minimum sample size of 335 was calculated with the Cochran’s formula using the estimated proportion of men involved in MCH as 32% from a study carried out in northern Nigeria [27]. Other variables used include standard normal deviate at 95% confidence (corresponding to 1.96), and the acceptable margin of sampling error (0.05). A total sample size of 418 was eventually utilized as the minimum calculated estimate was increased by 20% to increase precision.

### Sampling methodology

Multi stage sampling method was used to select the respondents. This was an appropriate method being a community-based study. The following stages were involved in the selection of the respondents:

**Stage 1-Selection of wards.** There are 11 wards in Oshodi-Isolo Local Government. Three wards were selected by simple random sampling (balloting) viz Oke-Afa, Isolo and Okota. Respondents were allocated equally to the three wards.
**Stage 2-Selection of streets.** A simple random sampling technique by balloting was used to select 10 streets per ward making a total of 30 streets.
**Stage 3-Selection of houses.** 14 houses were selected on each street by systematic sampling. But first, the houses per street were enumerated (numbered) to create a sample frame. The sampling interval (k) was calculated per street.
**Stage 4-Selection of households.** In most cases, a house was shared by multiple households living in different units. Only one household with an eligible male was required in each house. In houses with more than one household, a household was selected by simple random technique (balloting).
**Stage 5-Selection of respondents.** Only one respondent who met the inclusion criteria defined in the research was selected from the household. In places where there were more than one respondent, simple random sampling was done to select only one.

For the focus group discussion (FGD), 12 respondents were sampled purposively via face-to-face recruitment with the help of a community contact but 11 participated. The inclusion criteria used for the quantitative method as outlined earlier were also applied.

### Data collection

Quantitative data collection was done for a period of three months between October and December 2018 with the assistance of trained research assistants. A structured, pretested, interviewer administered questionnaire in English was used to obtain information on socio demographic characteristics, knowledge and involvement in MNCH and the barriers to male involvement. This was developed from several studies and documents [28–31]. (S1 Questionnaire) Some of the questions for knowledge include definition of antenatal care, services provided during antennal care, pregnancy danger signs and others. To assess involvement, respondents answered ‘Yes’ or ‘No’ to questions such as: ‘Did you accompany your wife for antenatal care services? Did you give consent to your wife to attend ANC? Did you ensure that breast milk was initiated early to your new born child after birth?’ The questions were in reference to the last child.

Qualitative data collection technique (FGD) was employed to obtain more information on the factors associated with male involvement in MCH. Twelve of the respondents were selected initially and invited but 11 of them honored the invitation and participated in the discussion. One person failed to attend due to other engagements. The discussion was held at a neutral venue in the study area where the selected respondents convened one week after the quantitative data collection. A semi-structured interview guide was used for the discussion and covered questions such as: ‘What roles do men play in pregnancy, delivery and afterwards in this community?’ ‘What are the difficulties men face in taking care of their wives/partners during pregnancy, delivery and after delivery?’ (S1 File) Questions were followed by probes and follow-up questions. The discussion lasted for 90 minutes and was tape-recorded. The FGD was moderated by one of the lead researchers, assisted by a time keeper and gate keeper. After the discussion, light refreshments and stipend for transportation were given to the respondents.

Employing the multistage sampling technique, pretesting of the study instrument and training of the data collectors were done to reduce any potential sources of bias.

### Data analysis

#### Quantitative

After the data was cleaned, each completed questionnaire was assigned a unique code and data were entered and analyzed using the EPI-INFO software version 7. Both descriptive and inferential statistics were employed in the study. The quantitative data generated from the study were summarized with mean and standard deviations. Inferential statistics were done, and the set level of significance was 0.05. Predictor variables for good knowledge and involvement in MCH were determined with multiple logistic regression.

To determine the level of knowledge of men about MNCH, each correctly answered question was scored 1, while an incorrect response was scored 0. The mean score was used as cut off point to categorize respondents into good and poor knowledge groups [29, 32].

To determine level of male involvement in MCH, questions with ‘Yes’ response indicates involvement and assigned a score of 1 while a ‘No’ response indicates non-involvement and was scored ‘0’. The mean score was used as cut off point to categorize respondents into good and poor involvement groups [29, 32].

#### Qualitative

For the FGD, the recorded discussion was transcribed verbatim and organized under various themes which were developed by a combination of deductive and inductive analysis [33]. Broad categories based on research questions and attribute codes were developed and further rounds of reading and analysis done to identify ways and factors influencing male involvement as seen in the community. Supporting quotes were selected and presented.

Ethical approval for this study no ADM/DCST/HREC/APP/2962 was obtained from the Health Research Ethics Committee (HREC) of the Lagos University Teaching Hospital (LUTH). Informed consent forms were signed by all participants prior to interview/discussion and confidentiality was maintained throughout the study.

## Results

Only nine respondents declined to be interviewed and they were replaced by balloting. A total of 418 questionnaires were administered, adequately filled and analyzed.

### Sociodemographic and family characteristics

Most of the respondents were between the ages of 31–40 years 232 (55.5%) [Mean ± Standard Deviation = 37.64±6.81years]. The majority were married 402 (96.2%), had secondary education 201 (48.1%), were of the Yoruba tribe 249 (59.6%) and Christians 258(61.7%). Virtually all the respondents were employed 414 (99.9%), most earned more than 75,000 Naira (208 USD) monthly 263(63.3%). Most of the respondents had one wife/partner 403(96.4%) and one to three children 314(75.1%). Slightly over half had one child below five years 223(55.3%). (Table 1).

**Table 1:** Socio demographic characteristics of respondents.

Variable	Freq (n = 418)	Percentage(%)
**Age of respondents (years)**		
21–30	72	17.2
31–40	232	55.5
41–60	114	27.3
Mean age = 37.64±6.81years		
Median age = 37.64 years		
**Marital status**		
Married	402	96.2
Cohabiting	16	3.8
**Years of married/ Cohabiting**		
1–5	192	45.9
6–10	166	39.7
>10	58	13.9
No Response	2	0.5
Mean = 6.85±4.22 years		
Median = 6.00years		
**Education**		
No formal/Primary Education	71	17.0
Secondary	201	48.1
Post secondary	146	34.9
**Tribe**		
Yoruba	249	59.6
Non-Yoruba	169	40.4
**Religion**		
Christian	258	61.7
Muslim	160	38.3
**Employment status**		
Employed	414	99.0
Unemployed	4	1.0
**Estimated monthly income (Naira)**		
30,000–50,000	94	22.9
50,001–75,000	57	13.9
>75,000	260	63.3
Non-Response	7	1.7
**Number of wives/partners**		
1	403	96.4
2	13	3.1
3	2	0.5
**Number of children**		
1	121	28.9
2	111	26.6
3	82	19.6
4	60	14.4
≥5	44	10.5
Mean = 2.56 ± 1.4		
Median = 2.00		
**Number of children below 5 years**		
1	223	55.3
2	165	39.5
≥3	30	7.2
Mean = 1.6 ± 0.66		
Median = 1.00		

Table 2 shows that most of the respondents’ spouses/partners were aged 25 years or older, had formal education and employed.

**Table 2:** Sociodemographic and economic characteristics of spouse/partner.

Variable	Freq (n = 418)	Percentage (%)
**Age of spouse (years)**		
18–24	26	6.2
25–29	120	28.7
30–34	154	36.8
34–47	118	28.2
Mean = 31.94 ± 5.36		
Median = 32		
**Education**		
No formal/Primary Education	42	10
Secondary	101	24.2
Post secondary	275	65.8
**Employment status of spouse**		
Employed	312	74.6
Unemployed	106	25.4
**Spouse income (Naira)**		
≤30000	23	7.4
30,001–75000	112	35.9
>75,000	177	56.7
Non-response	106	25.4

### Knowledge of maternal and child health care

The results on knowledge of maternity care are presented in Table 3. ANC services commonly known by the respondents were: physical examination 237(56.7%) and laboratory tests 231(55.3%). The most mentioned birth preparedness and complications readiness (BP/CR) components were ‘save money’ 297(71.1%) and ‘buy delivery kit’ 226(54.1%). The commonest obstetric danger signs identified by the respondents was severe vaginal bleeding 339 (81.1%) and for labour and delivery were: severe vaginal bleeding 259(62%) and prolonged labour lasting more than 12 hours 231 (55.3%). A few of the respondents knew the correct definition of post-natal care 166 (39.7%) while the post-natal danger signs most known include severe vaginal bleeding 245 (58.6%) and high fever 101 (24.2%).

**Table 3:** Respondents knowledge of maternity care.

Variable	Frequency (n = 418)	Percentage (%)
**Correct Definition of Antenatal care**	372	89.0
**Services provided during antenatal care** *		
Health education e.g. health talk	200	47.8
Physical examination	237	56.7
Laboratory tests	231	55.3
Preventive measures/therapy	158	37.8
Counselling	132	31.6
Treatment of existing illnesses	61	14.6
Others	16	3.8
Don’t know	19	4.5
**Advice given to a woman during antenatal care** *		
Better dietary Intake	303	72.5
Resting during the day	209	50.0
Intake of Iron Folic acid	63	15.1
Desisting from doing heavy work	211	50.5
Newborn care	81	19.4
Birth preparedness	32	7.7
Others	33	7.9
**The danger signs in pregnancy** *		
Severe vaginal bleeding	339	81.1
Swollen hands/face	83	19.9
Blurred vision	50	12.0
Others	49	11.7
Don’t know	13	3.1
**Things to be done in preparation for a woman’s delivery or emergencies** *		
Identify skilled attendant for delivery	208	49.8
Save money	297	71.1
Buy delivery kit	226	54.1
Arrange emergency transport	140	33.5
Arrangement for blood donor/donation	37	8.9
Others	20	4.8
Don’t know	2	0.5
**Knowledge of skilled birth attendant**		
Doctor	321	76.8
Nurse	291	69.6
Don’t know	15	3.6
Others (traditional birth attendant)	17	4.1
**Danger signs during labour/ delivery** *		
Severe vaginal bleeding	259	62.0
Prolonged labour (> 12 hours)	231	55.3
Convulsions	67	16.0
Retained placenta	61	14.6
Others	7	1.7
Don’t know	32	7.7
**Correct definition of Post Natal care**	96	23.0
**Danger signs during the post-partum/post-natal period (beginning immediately after delivery until six weeks)** *		
Severe vaginal bleeding	245	58.6
Foul smelling vaginal discharge	70	16.7
High fever	101	24.2
Others	79	18.9
Don’t know	75	17.9

*multiple responses

Table 4 shows respondents’ knowledge of newborn health and breastfeeding. Essential newborn care mainly mentioned by the respondents were ‘wiping baby with clean dry cloth’ 286 (68.4%) and cutting cord with sterilized instrument 192(45.9%). Regarding danger signs in the newborn, respondents knew convulsions/spasms/rigidity 147 (35.2%), lethargy/unconsciousness 108 (25.8%) and difficult/fast breathing 89(21.3%). About 90% knew that breastmilk is essential for a child’s growth and complimentary feeding should be introduced at six months 150(35.9%).

**Table 4:** Respondents knowledge of newborn health and breast feeding.

**Essential new born care**		
Wiping baby with clean dry cloth	286	68.4
Wrapping for warmth	103	24.6
Cutting cord with sterilized instrument	192	45.9
Initiation of breastfeeding within 1 hour of birth	76	18.2
Colostrum feeding	14	3.3
Others	12	2.9
Don’t know	7	1.7
**The danger signs in a new born within the first 7 days after delivery** *		
Convulsions/spasms/rigidity	147	35.2
Difficult/fast breathing	89	21.3
Very small baby	58	13.9
Lethargy/unconsciousness	108	25.8
Others	64	15.3
Don’t know	65	15.6
**Breastmilk essential for a child’s growth**	377	90.2
**Correct definition of exclusive breastfeeding**	297	71.1
**Duration of breastfeeding (2 years and beyond)**	103	24.6
**Age to introduce complimentary feeding (6 months)**	150	35.9

*multiple responses

Less than half of the men knew that ORS should be administered to a child with diarrhea and less than 10% knew about Zinc supplementation. Majority knew that a woman should continue breastfeeding a child suffering from diarrhoea. Only about 30% of the respondents knew the correct definition of immunization and the commonest immunizations mentioned were measles and yellow fever 208(49.8%) and poliomyelitis 206(49.3%). (Table 5).

**Table 5:** Respondents’ knowledge of child health (diarrhoea, ARI, immunization).

Variable	Frequency (n = 418)	Percentage (%)
**Danger signs of diarrhea in children** *		
Abdominal pain	156	37.3
Blood in stool	8	1.9
Frequent vomiting	132	31.6
Loss of appetite for liquids	61	14.6
High fever	121	28.9
Dry, sticky mouth	97	23.2
Weight loss	52	12.4
Frequent urination	72	17.2
Frequent stooling	200	47.8
Others (tiredness, nausea)	3	0.7
**Management of diarrhea in children** *		
Increased fluid consumption	173	41.4
Administration of ORS	183	43.8
Zinc supplementation	30	7.2
Increase of duration and frequency of breastfeeding in infants	60	14.4
Frequent feeding with nutritious and easily digestible foods	40	9.6
Others (herbal medicine, drugs)	26	6.2
Don’t know	35	8.4
**Correct knowledge of use of ORS in children**	189	45.2
**Continuation of breastfeeding during diarrhea (Yes)**	333	79.7
**Danger signs of acute respiratory infection (ARI) in children** *		
Cough	128	30.6
Sneezing	140	33.5
Runny nose	158	37.8
Nasal congestion	60	14.4
Fever	56	13.2
Itchy/sore throat	41	9.8
Nasal breathing	45	10.8
Others (swallowing difficulty, clenching teeth, painful breathing)	75	17.9
Don’t know	24	5.7
**Correct knowledge of definition of immunization**	127	30.4
**Knowledge of childhood immunizations**		
Measles & Yellow fever	208	49.8
Haemophilus influenza (Hib)	59	14.1
Polio vaccinations	206	49.3
Diphtheria, tetanus, and pertussis (DPT)	101	24.2
Hepatitis B	87	20.8
Rotavirus vaccine	59	14.1
Chicken pox	56	13.4
Others	20	4.8
Don’t know	8	1.9

*multiple responses, ARI- Acute respiratory illness

### Involvement in maternal and child health care

Most men gave their consent to attend antenatal care 359 (85.9%) and PNC 304(72.7%). Many also paid for the ANC 333(79.7%), delivery 357(85.4%) and PNC 277 (66.3%) but only few 122(29.2%) accompanied their wife to antenatal clinic at least once, or for PNC 132(31.6%). More of the respondents accompanied their wives for delivery 336 (80.4%).

Most of the men ensured early initiation of breastfeeding 285 (68.2%) and 274(65.6%) ensured that their new born received all recommended immunizations at birth. Almost two-thirds 269 (64.4%) had administered ORS to their children in cases of diarrhoea, and 354(84.7%) ensured that their children ate balanced diet. Only 106(27.5%) ensured that the children brushed their teeth regularly (Table 6).

**Table 6:** Respondents involvement in maternity care and newborn health.

Involvement in maternal health	(n = 418)	(%)
Attendance of wife/partner to antenatal care services when pregnant	354	84.7
**Decision for wife/partner to attend (or not) antenatal care was taken by:**		
Man	166	39.7
Wife/partner	193	46.2
Jointly with wife/partner	59	14.1
No response	1	0.2
Gave consent to wife to attend antenatal care	359	85.9
Accompanied wife to antenatal care at least once	122	29.2
Paid for antenatal care	333	79.7
**Decision on place of delivery was taken by**		
Man	173	41.4
Wife/partner	143	34.2
Jointly with wife/partner	91	21.8
Relative	6	1.4
Joint decision by man, wife and relatives	5	1.2
Non-Response	1	0.2
Accompanied wife/partner to delivery	336	80.4
Paid for delivery services	357	85.4
Gave consent to wife/partner to attend postnatal visits	304	72.7
Accompanied wife/partner for post-natal care services	132	31.6
Paid for the postnatal care services	277	66.3
**Newborn & child health care**		
Ensured that breast milk was initiated early after birth	285	68.2
Ensured that newborn received all recommended immunizations at birth	274	65.6
Present when baby’s birth weight was checked	182	43.5
Taken child to immunization before	237	56.7
Administered ORS to child when he/she had diarrhea	269	64.4
Ensured child started complimentary feeding at the right age	309	73.9
Fed children as infants till they were old enough to feed themselves	346	82.8
**Ensured child has a good hygiene by** * **:**		
-Bathing twice daily	312	80.8
-Brushing of teeth regularly	106	27.5
-Frequent washing of hands	181	46.9
-Wearing clean clothes	336	87.0
-Maintaining clean hair	133	34.5
Ensure child eats a balanced diet	354	84.7

*multiple responses

Table 7 shows that overall, 272(65.1%) had good knowledge while 146(34.9%) had poor knowledge. Also, 254(60.8%) had good involvement while 164(39.2%) had poor involvement in MCH.

**Table 7:** Overall level of men’s knowledge and involvement in maternal, newborn and child health.

Variable	Frequency (418)	Percentage (%)
**Knowledge**		
Good	272	65.1
Poor	146	34.9
Mean	80.3 ± 8.86	
Median	82.05	
**Involvement**		
High	254	60.8
Low	164	39.2
Mean	68.2 + 21	
Median	73.9	

The commonest barriers to involvement in MCH were: ‘lack of time’ 266(63.6%) and ‘culture and tradition’ 186(44.5%) as shown in Table 8.

**Table 8:** Barriers to male involvement in maternal and child health care.

Variable	Frequency (n = 418)	Percentage (%)
**Barriers to male involvement in MNCH** *		
Lack of funds	108	25.8
Culture and tradition	186	44.5
Attitude of others towards men involved in maternal and child health	134	32.1
Pride	101	24.2
Lack of time	266	63.6
Location of the hospital	35	8.4
Attitude of health workers	65	15.6
Others	16	3.8

*multiple responses

### Predictors of good knowledge and involvement in MCH

Predictors of good knowledge were being a Christian (AOR 1.674, 95% CI 1.045–2.679), being of Yoruba tribe (AOR 1.753, 95% CI 1.100–2.796), having post-secondary education (AOR 1.984, 95% CI 1.002–3.928), having more under-fives in the household (AOR 2.162 95% CI 1.365–3.425) and spouse having post-secondary education (AOR 2.755, 95% CI 1.189–6.382) (Table 9).

**Table 9:** Predictors of good knowledge of maternal, newborn and child health.

Predictor variable	p-value	AOR	CI
**Years cohabiting/married**	0.234	1.358	0.821–2.246
**Religion**			
Christianity	0.032	1.674	1.045–2.679
Muslim		1	
**Tribe**			
Yoruba	0.018	1.753	1.100–2.796
Non-Yoruba		1	
**Educational level of respondent**			
No formal and Primary		1	
Secondary	0.290	0.722	0.395–1.320
Post-secondary	0.049	1.984	1.002–3.928
**Estimated income of respondent**	0.842	1.000	1.000–1.000
**Number of children**	0.272	0.845	0.626–1.141
**Number of children <5 years**	0.001	2.162	1.365–3.425
**Educational level of spouse**			
No formal and Primary		1	
Secondary	0.234	1.635	0.727–3.675
Post-secondary	0.018	2.755	1.189–6.382
**Employment status of spouse**			
Unemployed		1	
Employed	0.082	0.596	0.332–1.069

AOR Adjusted Odds Ratio; SE Standard Error; CI Confidence Interval

Table 10 shows the predictors of good involvement in MCH which include higher educational level of spouse: secondary (AOR 2.852, 95% CI 1.214–6.699), post-secondary (AOR 2.270, 95% CI 1.000–5.161) and having good knowledge of MCH (AOR 2.518, 95% CI 1.587–3.994).

**Table 10:** Predictors of male involvement of maternal, newborn and child health.

Predictor variable	p-value	AOR	CI
**Years cohabiting/married**	0.990	1.003	0.586–1.719
**Tribe**			
Yoruba	0.002	0.466	0.289–0.751
Non-Yoruba		1	
**Estimated income of respondent**	0.002	1.000	1.000–1.000
**Number of children**	0.514	1.117	0.801–1.558
**Number of children <5 years**	0.092	1.475	0.938–2.319
**Age of Spouse**	0.148	1.040	0.986–1.097
**Educational level of spouse**			
No formal and Primary		1	
Secondary	0.016	2.852	1.214–6.699
Post-secondary	0.050	2.270	1.000–5.161
**Level of knowledge**			
Poor		1	
Good	<0.001	2.518	1.587–3.994

AOR Adjusted Odds Ratio; SE Standard Error; CI Confidence Interval

### Focus group discussion results

Table 11 shows some basic demographic information on the discussants. Their ages ranged from 30 to 60 years.

**Table 11:** Socio demographic characteristics of discussants.

Code no	Age (years)	Occupation	Ethnicity	Religion
1	30	Business man	Igbo	Christian
2	48	Civil Servant	Igbo	Christian
3	45	Truck Driver	Yoruba	Christian
4	39	Engineer	Yoruba	Muslim
5	39	Architect	Igbo	Christian
6	35	Teacher	Hausa/Fulani	Muslim
7	48	Commercial driver	Yoruba	Christian
8	54	Painter	Edo	Christian
9	38	Hairdresser	Yoruba	Muslim
10	42	Business man	Yoruba	Christian
11	60	Retiree	Yoruba	Christian

### Roles of men in pregnancy and delivery

A good number of the discussants felt that the roles of men in maternal care are limited to finance and transportation. These are seen as their traditional roles which they are limited to. They added that the men should ensure their wives attend antenatal care services but do not need to accompany them.

*“Men are the head of the head of the family*, *the dominant head*. *That means that anything finance related should channel from them*. *There is no need for a man to accompany a woman to the hospital for antenatal care unless he is jobless*. *But he must make the money available to ensure the woman doesn’t have any financial problems*. *He should also make sure the transportation needed to take her to the hospital whether for antenatal*, *delivery or emergencies is available*. *Many men like to be there during the delivery*, *I do not fault that but I don’t believe it is a role men have to play*.*”*

*(Civil Servant*,*48)*

However, very few discussants had contrary views saying the man is supposed to make it also a necessity to play more roles ie be more involved (like accompanying the woman to the health facility for maternity care) since the baby belongs to both of them. One of them argued vehemently that the men should be physically present for maternity care:

*‘My wife is currently pregnant and her antenatal days are on Friday*. *I take Friday morning off from work so that I can join her*. *I think we are too backwards in this country*. *The woman did not get herself pregnant so why should she be the only one attending antenatal*? *How will you even know how to take care of her or the baby or even the pregnancy risks and problems if you don’t join her*?*”*

*(Teacher*, *35)*

Another discussant also added that some men support their wives at home with the house chores and even cook and help the wife with personal hygiene. He gave a personal example:

*“A man is expected to support his wife when she is pregnant*. *Apart from the companionship that she will truly need in that period*, *He should cook for her*, *provide her cravings*, *wash for her*, *and even bathe her sometimes*. *I know my wife could not properly scrub her body when her belly became really big*. *You can’t expect her to do the same house chores she used to do when she was not pregnant; she is carrying your child*.*”*

*(Business man*, *30)*

#### Involvement of men with respect to the health of their children

Most discussants admitted that women usually play greater roles in personal hygiene and awareness of illness in children. The man’s role is usually in the provision of finance for medical treatment and routine medical care like immunizations.

*‘Most times mothers are the ones who spend more time with the children especially here in Nigeria*. *This doesn’t give a father the excuse to not be involved in the health of his child but you can’t deny that he has less influence in that area particularly because of time*. *So the mother is quick to notice signs of illnesses as well and she informs the father*. *He then should ensure the child receives proper medical attention’*

*(Retiree*, *60)*

*‘In many cases*, *it is the woman that sees to the personal hygiene of the child*. *Our mothers taught us everything we know*. *But nowadays*, *men are trying to get involved as much as they can*. *They are not usually patient enough*, *but they try*.*’*

*(Hair dresser*, *38)*

#### Difficulties men face in taking care of their wives/partners during pregnancy, delivery and after delivery

Some discussants opined that lack of knowledge of the intricacies, emotional changes and danger signs, which characterize the pregnancy period makes it difficult for some men to really take care of their wives. One of the men said;

“*You cannot take care of what you know nothing about*. *This is the reason why I believe men should accompany their wives to antenatal care*. *Some men are clueless when it comes to the things that should be looked out for during and after pregnancy*. *I was that way when my wife was pregnant with our first child*. *It was difficult for me because every complaint made me anxious*, *calling friends and relatives and even booking appointments with doctors’*.

*(Business man*,*42)*

Taking care of a pregnant woman was also seen as time consuming and expensive hence the need to work harder leaving little time for involvement in MNCH. With respect to delivery, the men reported that many of the hospitals do not permit the man into the labor room and so limiting their involvement.

### Factors that influence male involvement in maternal and child health (barriers, facilitators)

#### I. Barriers

The discussants presented different perspectives with respect to some of the factors that prevent them from participating fully in MCH care viz: socio-cultural beliefs, time constraint, stigmatization of men perceived to be ‘involved in MCH care’, health workers attitudes and costs associated with maternity care.

*Socio-cultural beliefs*. According to the discussants, it is culturally believed that things that concern MCH care are for women and do not concern men:

“*Cultural beliefs still have a strong hold on Nigerian men*. *The ill assigned gender roles when it comes to maternal and child care completely excludes the man from doing anything except provide money*, *security and shelter for the wife and child*. *How can a man be unable to bathe or feed his child*? *They believe it is a woman’s affair*. *This is wrong”*

*(Architect*, *39)*

*Time constraint*. According to some of the discussants, most times because of work, men are constrained, and their unavailability limits their involvement in the health of their wife and children;

“*To earn money*, *a man needs to work*, *this same time is what he will need to take care of his wife and children*. *Many men do not have that luxury*. *The absence of the man means he will be les involved during pregnancy*, *delivery and afterwards*. *And if he doesn’t work*, *there’ll be no money for the woman to even take care of herself”*

*(Commercial driver*, *48)*

*Health worker attitudes*. Few of the discussants reported that health worker attitudes discourage men from becoming involved:

*”There is something about the attitude of some of the health service providers*, *especially the older ones*. *They are of the opinion that the antenatal care and delivery is the women’s arena and they regard the men who accompany women to antenatal in a certain derogatory way"*.

*(Business man*, *42)*

However, some others disagreed, saying that staff attitude was not usually the issue emphasizing that some medical staff are committed to ensuring that men participate in maternal health to the extent of even organizing health education programs to enlighten men on the need to be more involved in MCH.

*Financial capacity of the man*. “*A man who is financially stable does not need to be all over the place looking for money to take care of his wife and children*. *A financially free man is more available*, *more at ease and more eager to care for his wife and children’*

*(Civil servant*,*48)*

According to another respondent, the man will make less money if he spends so much time attending antenatal care and staying home to take care of the children. *“This is sacrifice*, *a very necessary sacrifice*.*’(Painter*, *54)*

*Family background*. Some discussants added that a man’s up-bringing can also be a barrier to his involvement.

*“Some men never grew up to see their fathers participating in anything that had to do with maternal or child care*. *This shaped their ideologues and behaviors”*

*(Engineer*, *39)*

#### II. Facilitators

Facilitators of male involvement refers to those factors that reinforce men’s involvement in maternal and child health.

*Knowledge of the benefits of male involvement in maternal and child health*. One of the main facilitators mentioned during the focus group discussion is men’s knowledge of the benefits of their involvement in maternal and child health:

*“When you see the positive results of things you do*, *you will be encouraged to do even more*. *When a man is involved in his wife’s and child’s health*, *there is an obvious positive difference and any reasonable man will know that*. *If his friends are observant enough they will notice it and even try to emulate it; it’s all about recognizing the impact of your involvement as a man”*

*(Architect*,*39)*

*Orientation and advocacy*. Some of the discussants reported that health and gender messaging/advocacy and orientation play an important role in encouraging men to participate in maternal and child health.

*“If the health sector really believes in the advantages of male involvement*, *then they should make some noise about it*. *Things like these have worked in the past*. *Make radio jingles*, *carry out trainings and orientations*, *just an all-round education of the men so that it is ever fresh in their minds and it will even become abnormal for a man to not be involved in maternal and child*.*’*

*(Retiree*, *60)*

Some discussants mentioned that gender focused messages in the past usually by religious institutions has made men realize that they need to take a more active roles in household chores.

## Discussion

Men in this study were fairly knowledgeable about MCH while three-fifths had good involvement especially in the area of finance, consent and decision-making. The identified predictors of male involvement in MCH were higher educational level of spouse and non-Yoruba tribe. Other factors as learned from the FGD include cultural beliefs and tradition, health system and lack of time.

Similar to a research carried out in Bangladesh, there was a high knowledge of the services and advice provided during ANC [34] There was however, a lower proportion of the respondents who knew that newborn care and birth preparedness were part of the advice given during ANC. These gaps need to be covered to improve men’s knowledge and involvement in MCH.

Severe vaginal bleeding has been widely mentioned by men as a danger sign in pregnancy [23, 27, 34]. The same applies to our study. This is possibly because it is a very visible sign compared to some other signs and also the commonest cause of maternal death [35]. Other obstetric and delivery danger signs were barely mentioned. This might pose real danger with respect to the woman should any of these complications arise, resulting in delay in seeking care and higher risk of maternal death. Hence the need for men’s education on obstetric and newborn danger signs.

With regards to knowledge of BP/CR, less than one in ten of the respondents knew there was need to arrange for blood donor/donation, yet they knew about the danger of obstetric haemorrhage. This poor knowledge was also reported in Kano, northern Nigeria where only 0.8% of the respondents considered it a need [27]. This knowledge gap is dangerous considering that haemorrhage is the commonest cause of maternal death. A potential blood donor and a decision maker need to be identified as every pregnant woman faces the risk of sudden, unpredictable complications that could end in death or injury to her or to her infant [31]. BP/CR is a strategy designed to reduce maternal mortality and men as the major decision makers should have good knowledge of it and ensure that they are ready ahead of delivery.

Diarrhoea is one of the five leading causes of death in children under five but men in this study had poor knowledge of it just like in Guinea Bissau [36]. Discussants in the FGD believed that men usually know very little about such issues. They were only meant to ensure that the child receives appropriate treatment when ill. A paradigm shift in gender dynamics will propel them to take interest and know more about MNCH. This may be achieved by gradual re-orientation of males through the traditional/cultural and religious systems, health workers educating men in the community on MNCH, in addition to early awareness creation for boys in schools.

Overall, respondents were not knowledgeable about obstetric, delivery and newborn danger signs though they had high educational level. This may limit the necessary support given to women by their male partners during pregnancy, delivery and afterwards. The importance of having good knowledge is necessary in reducing maternal mortality by preventing delay in seeking care. One discussant in a similar study noted that what men do not know can hurt women’s health [37]. There was a gap between knowledge and involvement. The involvement in this case, particularly with maternal care had a higher focus on consent, decision making and financing. They had low rates of physical presence in maternity care activities. This was corroborated in the FGD where it was said that provision of finance and transportation were seen as the traditional roles of men. Such deep-rooted belief common in patriarchal settings should be an avenue for health workers to involve traditional institutions at the community level in the education of men to improve their involvement. Authors around the world made similar observations [38–42]. Other studies in the country also show that men are more involved in the financial aspect of maternity care which culturally and traditionally is the major role of the man more than other areas [20, 23, 27, 43]. Irrespective of these norms, there is the need for the men to accompany their wives to ante and post-natal care services and interventions need to be put in place for this to happen. Community meetings, peer education and one-on-one counseling are effective strategies in engaging men [44].

Christians, Yorubas and those who had more under-fives in the household were about two times more likely to have better knowledge. The presence of multiple young children confers experience which likely increased their knowledge. Higher educational level of men and spouse also predicted good knowledge. The role of higher educational level in knowledge was also reported by other authors [27, 45]. Education exposes individuals to a lot of information and broadens their knowledge. As would later be brought up in the FGD, religion and culture were important in male knowledge and involvement in women’s and children’s health. These institutions should be targeted in design and implementation of interventions. Social media use is another predictor reported in East Africa [32]. Higher educational level of spouse and non-Yoruba tribe also predicted better male involvement among our respondents. In northern Nigeria, ethnicity also influenced involvement [29]. This buttresses the importance of formal education and further highlights the role of culture in male involvement in MCH. Authors have reported that reading newspaper, formal education of the man [46], older age of the man (40-49years) and social media use [32] predict active involvement. Predictors of lower involvement in other studies include lower empowerment of spouse and increased maternal age at marriage [47].

Some factors that influence male involvement in MCH were identified in the qualitative studies as well as the quantitative studies, some were barriers and others, facilitators/enablers. Major barriers were socio-cultural beliefs and time constraints (from both quantitative and qualitative aspects). Our patriarchal system seems to limit men’s involvement in MCH care to consent as major decision makers and financial provision. Religious and cultural factors and time constraint as major barriers to involvement have been previously reported [19, 29, 30, 32]. Other barriers such as pride, poor attitude of health workers, lack of funds leading to opportunity cost, among others have been identified in other studies [30, 36, 39–42, 48]. In some places, women seem to support this cultural exclusion as they do not want their male partners accompanying them [38, 39]. These barriers can be reduced to the minimum with focused and consistent intervention programs involving gender messaging (which can be driven mainly by religious leaders), advocacy and health education as suggested by our discussants. Use of men as change agents and community health workers to educate men have been effective in improving male knowledge and involvement in MCH [40, 41, 44]. This strategy should be implemented in the study setting and the important role of religious and traditional leaders recognized and utilized. Training of health workers on interpersonal communication is also vital to improve the participation of men. The clinic centered factors that hinder male participation in maternal and child health in the area of study can be addressed by making clinics more ‘father-friendly’. This requires the health service providers to have a more welcoming attitude towards fathers attending the clinic and to be mindful of the needs of fathers. Since the research found that higher educational level of the spouses predict good knowledge and involvement, stakeholders can put more efforts into education of the girl child to go beyond primary school level.

### Strengths and limitations

The numerous variables covered in our study constitutes a rich addition to the body of knowledge on this vital topic and may explain why despite a lot of resources expended, progress in maternal and child death reduction is slow in similar settings. The mixed method approach enriched the study, better insight into issues surrounding male involvement were brought to fore. Rigorous methodologies were followed, and validity of data collected were ensured throughout the study. The large sample size and community-based nature of the study provide current observations at the grassroot level which is important for programme planners, policy makers and all stakeholders in MCH. The cross-sectional design limits causal inferences and the face-to-face interviews may influence some responses especially with regards to involvement. Responses are ‘as reported’ and not verified and the possibility of recall bias cannot also be ruled out. Since a representative sample was used for the quantitative study, less emphasis was placed on the qualitative aspect which involved one FGD session. Limited resources did not permit for more sessions to be carried out thus saturation may not have been achieved.

## Conclusion

The men had knowledge gaps in obstetric and delivery danger signs as well as newborn and postnatal care. Male involvement centred mainly around finance, consent and decision making and was predicted by higher educational level of spouse and better knowledge of MCH. Other factors revealed in the FGD include cultural beliefs and tradition, religion, health system attributes and time constraint.

Community-based enlightenment programs can be carried out to stress the need for involvement of men in promoting MCH and also being agents of change in improving the quality of life of women and children. The men should be engaged in gender-transformative work to change their patriarchally informed notion that they should be decision-makers when it comes to their partners’ engagement in RH services including MCH. This re-orientation can be enhanced by inclusion of responsible parenthood in family life education for school boys in anticipation of their future roles as spouses and also involving traditional and religious institutions.

## Supporting information

S1 Questionnaire (DOCX)

S1 File Interview guide for focus group discussion. (DOCX)
