# Supplementary material for: “Men are the head of the family, the dominant head”: A mixed method study of male involvement in maternal and child health in a patriarchal setting, Western Nigeria
Source: PLoS One. 2022 Oct 26;17(10):e0276059. doi: 10.1371/journal.pone.0276059 (PMC9604958; doi:10.1371/journal.pone.0276059)
Supplement: S1 Questionnaire — (DOCX) [file pone.0276059.s001.docx]

**QUESTIONNAIRE**

**A mixed method study of male involvement in maternal and child health in a patriarchal setting, Western Nigeria.**

**SECTION A**

**Socio Demographics and characteristics of respondents**

1. Age (years) __________
2. Marital status – Married [ ] Cohabitiing [ ]
3. How long have you been married/Cohabiting? __________
4. What is the highest educational level you have attained?

No formal education: [ ] Primary education: [ ]
Secondary education: [ ] Post-secondary [ ]

1. Religion

Christianity: [ ] Islam: [ ]
Others (Please specify)____________

1. What tribe are you from?

Yoruba [ ] Igbo [ ] Hausa [ ] Others (Please state)________

**Economic Characteristics**

1. Employment Status - Employed [ ] Unemployed [ ]
2. Occupation ____________
3. Estimated monthly income/allowance ____________________

**Family Characteristics**

1. Number of wives ______
2. Number of children______
3. Number of children below 5 years_____

**Characteristics of spouse/partner**

1. Age of spouse/partner_______
2. Educational level of spouse/partner

No formal education: [ ] Primary education: [ ]

Secondary education: [ ] Post-secondary [ ]

1. Employment status of wife/partner

Employed [ ] Unemployed [ ]

1. Spouse/partner’s estimated monthly income ____________

**Information on the index child**

1. Age as at last birthday ………. Years
2. Gender a. Male [ ] b. Female [ ]
3. Birth order in the family ………

**KNOWLEDGE ON MATERNAL, NEONATAL AND CHILD HEALTH**

**Knowledge on Maternal Health Care**

1. What is Nigeria’s legal age for a girl to get married? ........................
2. At what age is a girl suitable to conceive? .................................
3. Are you aware women need special care? Yes [ ] No [ ]

**Prenatal care**

1. Do you know about antenatal care? Yes [ ] No [ ]
   a. What is antenatal care (tick one option)
   - - 1. Antenatal care is the care a woman receives during pregnancy given by a trained by a trained birth attendant for best possible outcomes for women and newborns
       2. Antenatal care is the care a woman receives after pregnancy
       3. Antenatal care is the care a woman receives during pregnancy to enable her rest well
       4. Antenatal care is the care given by traditional birth attendants during delivery.
2. How many times (minimum) should a pregnant woman attend antenatal care?.............
3. What are the Services are provided during antenatal care?

 Health education e.g. health talk
 Physical examination
 Laboratory tests
 Preventive measures/therapy
 Counselling
 Treatment of existing illnesses
 Others_______________________________

 Don’t know

1. What are the advice given to a woman during antenatal care(Multiple answers)

 Better Dietary Intake
 Resting during the day
 Intake of Iron Folic acid
 Not doing heavy work
 New born care
 Birth preparedness

 Others (please specify) ___________________

 Don’t know

1. What are the danger signs in pregnancy that you know?

 vaginal bleeding
 convulsions/fits
 severe headaches with blurred vision
 fever and too weak to get out of bed
 severe abdominal pain
 fast or difficult breathing.
 Others____________________
 Don’t know

**Delivery**

1. What do you need to do in preparation for a woman’s delivery or emergencies? (You can tick more than one option)

 Determine skilled attendant at delivery
 Save money
 Buy delivery kit
 Arrange emergency transport
 Arrangement for blood donor/donation
 Others ___________________
 Don’t know

1. Who is a skilled birth attendant? (Multiple answers)

 Doctor Nurse  Don’t know
Others (Please specify)_______________

1. What are the danger signs during labour/ delivery?
    Severe bleeding
    Severe Headache
   Convulsions
    Labour lasting more than 24 hours
    Placenta not delivered in 30 minutes after delivery
    Others (please specify) ______________________
    Don’t know

   **Post natal**
2. What is post-natal care? (Tick correct answer)
    Care given to the new born child immediately after birth
    Care given to the mother immediately after birth
    Care given to the mother and newborn baby immediately after birth
    Care given to the mother and newborn baby immediately after birth and for the first six weeks of life
    Others (please state)___________________
   Don’t know
3. What are the danger signs in the two days post-natal in women? (You can tick more than one option)

 Increased vaginal bleeding
 Fits
 Fast/difficult breathing
 Severe headaches with blurred vision
 Calf pain, redness or swelling; shortness of breath or chest pain
 Fever and too weak to get out of bed
 Others___________________________
Don’t know

**Newborn Health**

1. What are the things that should be done to a baby in the health centre immediately he/she is born?

 Wiping baby with clean dry cloth
 Wrapping including head
 Cutting cord with sterilized thread
 Tying cord with sterilized thread
 Initiation of breastfeeding within 1 hour of birth
 Colostrum feeding
 Others (Please specify)
 Don’t know

1. What are the danger signs in a new born within 7 days of delivery (you can tick more than one option)

 Difficulty in breathing or in-drawing
 Fits
 Fever
 Bleeding
 Not feeding
 Yellow palms and soles of feet
 Diarrhoea
 Others (Please specify)______________
 Don’t know

**Child Healthcare**

1. What are the danger signs of diarrhea in children?- You can tick more than one option

 Abdominal pain
 Blood in stool
 Frequent vomiting
 Loss of appetite for liquids
 High fever
 Dry, sticky mouth
 Weight loss
 Frequent urination
 Frequent stooling
 Extreme thirst
 No tears when crying
 Depressed fontanelle-soft spot-on infant’s head

Others (please specify)………………………………………………………………………

1. How can diarrhea be managed in children? (multiple options)

    Increased fluid consumption
    Administration of ORS
    Zinc supplementation
    Increase of duration and frequency of breastfeeding in infants
    Frequent feeding with nutritious and easily digestible foods
    Others (please specify) ________________
    Don’t know
2. What is ORS used for in children- Pick one option

 Bathing
 Feeding
 Treatment of malaria
 Prevention/treatment of dehydration due to diarrhea
 Others (Please specify)
 Don’t know

1. Should a mother continue breastfeeding a child during diarrhea? Yes [ ] No [ ]
2. Is breastmilk essential for a child’s growth? Yes [ ] No [ ]
3. At what age should a child be taken off breastfeeding? ___________
4. At what age should a child be introduced to complimentary feeding? _______
5. What do you understand by exclusive breastfeeding?
6. Acute respiratory infection is a serious infection that prevents normal breathing function.

What are the danger signs of this infection in children? You can pick more than one option

 Cough
 Sneezing
 Runny nose
 Nasal congestion
 Fever
 Scratchy or sore throat
 Nasal breathing
 Others (Please specify)______________
 Don’t know

1. What is immunization?

 Giving injections to treat illnesses
 Giving a vaccine to a person to protect them against diseases
 Going a person the remedy to a problem
 Injection given to babies to grow

1. What are the childhood immunizations that you know

    Measles, mumps, and rubella (MMR)
    Haemophilus influenza (Hib),
    Polio vaccinations (IPV)
    Diphtheria, tetanus, and pertussis (DPT)
    Hepatitis B
    Rotavirus vaccine
    Chicken pox
    Others (please specify )____________
    Don’t Know

**INVOLVEMENT IN MATERNAL & CHILD HEALTH
Maternal Health**

1. Are men supposed to accompany their wives to assess maternal health care? Yes [ ] No [ ].
2. Did your wife/partner go for antenatal care services when she was pregnant? Yes [ ] No
3. Who decided that she attend antenatal care?
   Your Decision
   Your wife/partner
    Jointly with your wife/partner
    Relative
    Joint decision by you, your wife/partner and relatives
4. Did you give consent to your wife to attend antenatal? Yes [ ] No [ ]
5. Did you accompany your wife for antenatal care services? Yes [ ] No [ ]
6. Did you pay for the antenatal care services? Yes [ ] No [ ]
7. Who decided where your wife would deliver?
    Your Decision
    Your wife
    Jointly with your wife
    Relative
    Joint decision by you, your wife and relatives
8. Did you accompany your wife to the hospital for delivery? Yes [ ] No [ ]
9. Did you pay for the delivery services? Yes [ ] No [ ]
10. Did you give consent to your wife/partner to attend postnatal visits? Yes [ ] No [ ]
11. Did you accompany your wife/partner for post-natal care services? Yes [ ] No [ ]
12. Did you pay for the postnatal care services? Yes [ ] No [ ]

**Newborn Health**

1. Did you ensure that breast milk was initiated early to your new born child after birth
   Yes [ ] No [ ]
2. Did you ensure that your new born received all recommended immunization for newborns within hours of birth Yes [ ] No [ ]
3. Were you present when your child’s birth weight was checked Yes [ ] No [ ]

**Child healthcare**

**61.** Have you taken your child to immunization before? Yes [ ] No [ ]

**62.** Have you administered ORS/T to your child when he/she has diarrhea? Yes [ ] No [ ]

63. Did you ensure you child started complimentary feeding at the right age Yes [ ] No [ }

65. Did/do you serve your children food as infants till they were old enough to feed themselves
Yes [ ] No [ ]

66. Do you ensure your child does the following? (good hygiene)

 Bathing twice daily
 Brushing of teeth regularly
Occasional washing of hands
Wearing clean clothes
 Maintaining clean hair

67. Do you ensure your child eats a balanced diet Yes [ ] No [ ]

**BARRIERS TO MALE INVOLVEMENT IN MATERNAL AND CHILD HEALTH CARE**

68. Mention some barriers to male involvement in maternal, newborn and child health care (Multiple answers)

 Lack of funds
 Culture and tradition
 Attitude of others towards men involved in maternal and child health
 Pride
 Lack of time
 Location of the hospital
 Attitude of health workers
 Others­­­­­­­­­­­­­­­________________
