# Supplementary material for: “Men are the head of the family, the dominant head”: A mixed method study of male involvement in maternal and child health in a patriarchal setting, Western Nigeria
Source: PLoS One. 2022 Oct 26;17(10):e0276059. doi: 10.1371/journal.pone.0276059 (PMC9604958; doi:10.1371/journal.pone.0276059)
Supplement: S1 File — (DOCX) [file pone.0276059.s002.docx]

*“Men are the head of the family, the dominant head”***: A mixed method study of male involvement in maternal and child health in a patriarchal setting, Western Nigeria**

**INTERVIEW GUIDE FOR FOCUS GROUP DISCUSSION**

1. What roles do men play in pregnancy, delivery and afterwards in this community?

-Follow up questions with specific examples

- Probe for any contextual issues

1. From your observation, what are the roles of men in childcare/health in this community?

- Follow up questions with specific examples

-Probe for any contextual issues

1. What are the difficulties men face in taking care of their wives/partners during pregnancy, delivery and after delivery? Can you give me any examples?

-Probe for specific examples and explanations

1. What limits men in being involved in issues concerning pregnancy, childbirth and postnatal care? (barriers, limitations)
2. What about the children? What limits male involvement in childcare/health?
3. Repeat questions 4 & 5 for ‘facilitators, enablers’
